# Study on Equity and Efficiency of Health Resources and Services Based on Key Indicators in China

**DOI:** 10.1371/journal.pone.0144809

**Published:** 2015-12-17

**Authors:** Xinyu Zhang, Lin Zhao, Zhuang Cui, Yaogang Wang

**Affiliations:** 1 Department of Health Service Management, School of Public Health, Tianjin Medical University, Tianjin, China; 2 Department of Epidemiology and Health Statistics, School of Public Health, Tianjin Medical University, Tianjin, China; Kenya Medical Research Institute - Wellcome Trust Research Programme, KENYA

## Abstract

**Background:**

This study aims to evaluate the dialectical relationship between equity and efficiency of health resource allocation and health service utilization in China.

**Methods:**

We analyzed the inequity of health resource allocation and health service utilization based on concentration index (CI) and Gini coefficient. Data envelopment analysis (DEA) was used to evaluate the inefficiency of resource allocation and service utilization. Factor Analysis (FA) was used to determine input/output indicators.

**Results:**

The CI of Health Institutions, Beds in Health Institutions, Health Professionals and Outpatient Visits were -0.116, -0.012, 0.038, and 0.111, respectively. Gini coefficient for the 31 provinces varied between 0.05 and 0.43; out of these 23 (742%) were observed to be technically efficient constituting the “best practice frontier”. The other 8 (25.8%) provinces were technically inefficient.

**Conclusions:**

Health professionals and outpatient services are focused on higher income levels, while the Health Institutions and Beds in Health Institutions were concentrated on lower income levels. In China, a few provinces attained a basic balance in both equity and efficiency in terms of current health resource and service utilization, thus serving as a reference standard for other provinces.

## Introduction

Equity and efficiency in health resource-allocation and health service utilization is being increasingly advocated [1, 2]. The concept of equity in health care has been widely debated over the years. Although equity may be defined in several ways, it implies a fair distribution of an entity such as health services among different individuals and groups in the society [3]. Equity in health resources is divided along horizontal and vertical dimensions [4]. The World Health Organization advocates the concepts of horizontal equity, healthcare to those in primary health need, and vertical equity, addressing those with the greatest need [5]. Health efficiency is a measure of output (such as hospital business income, outpatient visits, and bed utilization rate) a province can achieve using a certain level of input (such as health institution, health professionals, beds in health institutions, and total health expenditure). Efficiency studies help to delineate areas where costs could be reduced and output increased for a more efficient utilization of health services [6]. It is a means to provide access to basic health services equitably and effectively, and ultimately leading to improved health status.

**Fig 1:**
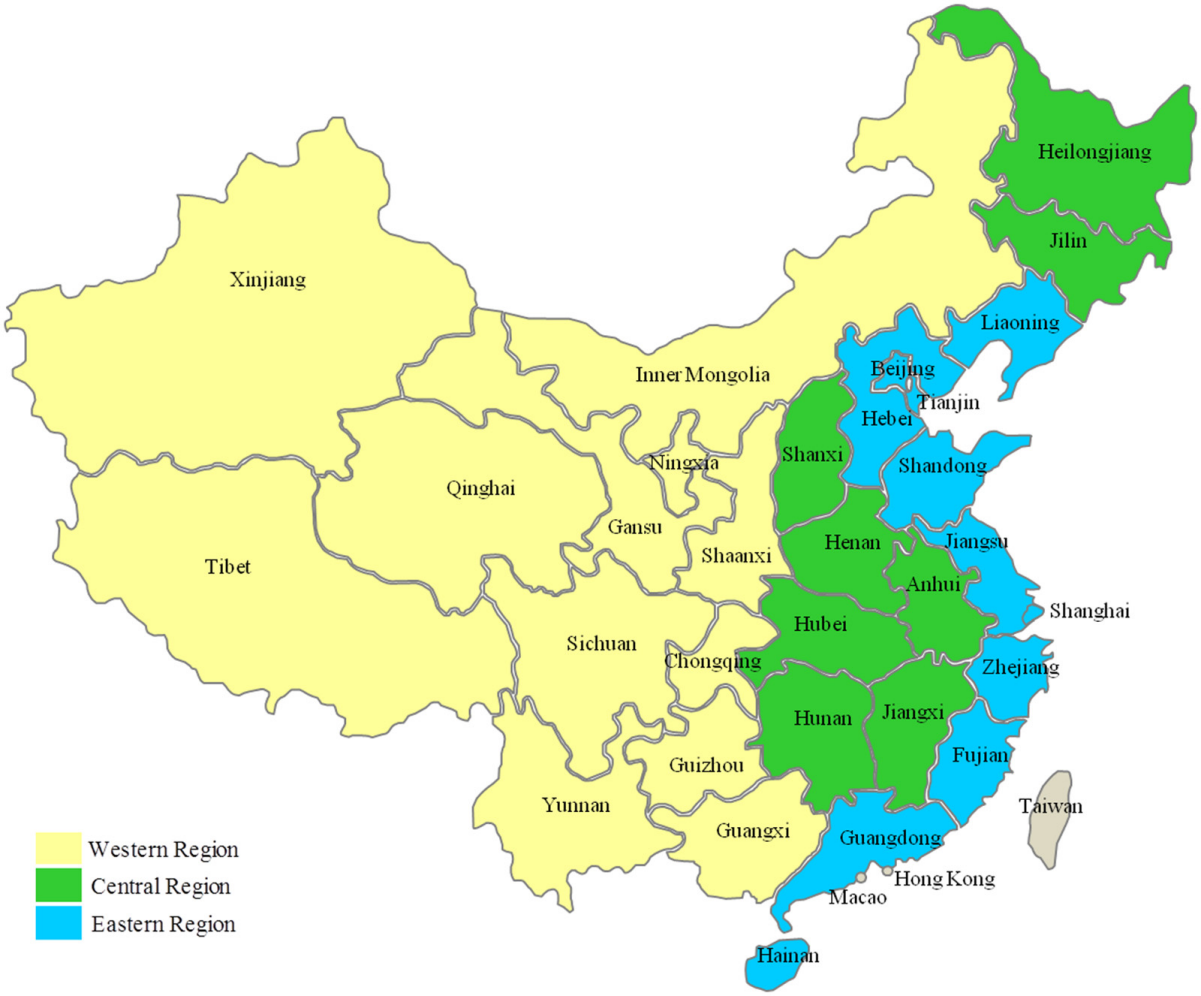
The eastern, central and western regions of China.

China has been undergoing the most rapid industrialization and urbanization in recent decades. Due to the diversity in Chinese geography, there is an imbalance in economic development between the eastern, central, and western regions, causing inequity and inefficiency in health resources allocation. The western and central regions lack high quality health resources rendering it difficult to access health care. The high cost of medical services is significant barrier that prevents access to health services (Fig 1). The eastern, central, and western regions of China are divided according to regional economy, population, environment, and other factors. In contrast to the prosperous eastern region, and the industrial and agricultural central region, the western region, situated inland is environmentally and economically underdeveloped. The humid climate in the eastern and central plains is more suited to agriculture and animal husbandry, while the rugged terrain in the west, with its mountains and plateaus, contributes to inclement weather. In terms of population, the eastern region covers a small but densely populated geographical area. The central region is mainly rural, while the western region is geographically vast but sparsely populated (S1 Table).

Equity and efficiency in health resource allocation and service utilization are important objectives for the healthcare system that are increasingly attracting attention in China [7]. The Chinese government has revised its approach to improve equity and efficiency of health resource and services [8–11]. In this paper, we used concentration index (CI), Gini coefficient and data envelopment analysis (DEA) to evaluate the equity and efficiency in health resource allocation and health service utilization in the 31 provinces of China.

## Methods

### Ethics Statement

All participants provided written informed consent to participate in this study as well as for publishing the case details. The study was approved by the institutional review board and the ethics committees at the Tianjin Medical University, Tianjin, People’s Republic of China.

### Methods of equity evaluation

The CI and the Gini coefficient have been identified as superior tools for measuring inequity in health resource allocation [12]. Concentration index allows for assessment of the distribution of health resources by economic status of health care beneficiaries, and reflects the relationship between health allocation and family income. The Gini coefficient examines the distribution of health resources in the total population, and reflects the relationship between allocation and population density.

In this study, CI is used as a measure of inequity in health resource allocation by health indicators. The Gini coefficient is used a measure of the inequity in health resource allocation between provinces.

#### Concentration index

A concentration curve was drawn with the cumulative proportion of a health outcome on the y-axis, against the cumulative population ranked by economic status of the beneficiaries on the x-axis [13]. If everyone in a given population, irrespective of economic status, had exactly the same health outcome, the concentration curve would be represented by a 45° line running from the bottom left-hand corner to the top right-hand corner of the plot. The CI is defined as twice the area between the concentration curve and the line of equality [14]. The value of CI ranges from −1 to 1, with 0 indicating perfect equality. The index is negative if it lies above the line of equality, which indicates a disproportionate concentration of the variable among individuals with lower income levels. It is positive if it lies below the line of equality, indicating that the variable was disproportionately concentrated among individuals with higher income levels. The greater the distance between the concentration curve and the line of equality, the more concentrated are the health variables among individuals with lower or higher income levels [15].

The concentration index is represented by the following equation [16]:

$$\text{C}=\frac{2}{\mu }\text{cov}\left(h,r\right)$$

where *h* is the health variable, *r* is the fractional rank in terms of household economic status, and *μ* is the mean of the health variables.

#### Gini coefficient

In this study, the Gini coefficient was adopted as a measure of the inequality in health resource allocation between provinces. The Gini coefficient is calculated as the ratio of the area between the Lorenz curve and the diagonal line, to the whole area below the 45° line. The following formula was used to calculate the Gini coefficient:

$$G=\left|1+{\sum^{\text{​}}}^{\text{​}}FnPn-2{\sum^{\text{​}}}^{\text{​}}{\left({\sum^{\text{​}}}^{\text{​}}Pn\right)}^{\prime }Fn\right|$$

where *G* is the Gini coefficient, *Fn* is the proportion of the shifted integral factor score to the sum of all regional scores, *Pn* is the proportion of the regional population in the total population, and (∑*Pn*)′ refers to the cumulative proportion in the total population.

### Efficiency evaluation

#### Data envelopment analysis (DEA)

Data envelopment analysis (DEA), a nonparametric mathematical programming methodology first developed by Charnes *et al* [17] uses the frontier approach to measure the relative efficiency or performance of decision-making units (DMUs), based on a fractional programming problem that has been converted into a linear programming problem. The efficient DMUs that represent the “best practice frontier” are assigned an efficiency score of 1. The inefficient DMUs are assigned a score between 1 and 0 [18]. Charnes *et al* developed the first model as the constant return to scale (CRS) model, which was used to measure each DMU's Overall Efficiency (OE), whether the "technology and scale" was effective at the same time. R.D. Banker, A. Charmes, and W.W. Cooper developed the variable return to scale model (VRS model) in 1984, which is used to measure the technical efficiency of DMU with altered size of return [19]. VRS model divided the OE of the CRS model into technical efficiency (TE) and scale efficiency (SE). The VRS model assumes that each DMU scale return may increase, decrease, or remain unchanged.

Overall efficiency was measured using the CRS model, which refers to similar circumstances and actual output as the ratio of the maximum output. TE and SE were measured using the VRS model. Technical efficiency is defined as the ratio of the minimum (optimal) amount of input to the actual input levels of a DMU for a given level of output, keeping the input proportion constant [20]. Scale efficiency refers to the constant evaluation of the scale return, the multiple output increase as equivalent to increase in multiple inputs. Overall efficiency is calculated using the following formula:

OE = Technical Efficiency * Scale Efficiency

The TE and SE scores were computed using the DEA Program, version 2.1 (DEAP 2.1), developed by Tim Coelli. DEA model was tested for robustness.

#### Data and variables

Our study was focused on health resource allocation and health service utilization in specific areas rather than in an individual hospital or institution. Data were obtained from the national public databases: the China Statistical Yearbook and the Annual National Health Report. Data on the socioeconomic status of provinces was sourced from the China Statistical Yearbook. The health resource and health survival data were sourced from the Annual National Health Report for 2012 issued by the Chinese National Health and Family Planning Commission. The study was partly based on a national representative survey dataset. The study data were derived from the same time span and statistical unit as the database. In this research, we considered every province as an analytical unit. The health resources and services in the 31 provinces, autonomies, and municipalities of China were studied. The province represents the unit of analysis. Population, socioeconomic and health resources in each province were computed at the micro level (City, Town, County or District) previously. The use of geographic units as DMUs in the DEA method is based on a previous study [21]. A total of 31 DMUs were used to reflect the regional administration according to Chinese geography.

Similar to the other statistical methods, stability in the form of degrees of freedom is also an issue in DEA. In this method, the degrees of freedom increased with the number of DMUs, and decreased with the number of inputs and outputs. A general rule of thumb is [22]:

n≥max{m×s,3(m+s)},

where *n* represents the number of DMUs, *m* denotes the number of inputs, and *s* refers to the number of outputs. Therefore, the minimum number of DMUs is either the product of the number of inputs and the number of outputs, or three times that of the sum of the number of inputs and outputs, whichever is bigger [23].

The indicators in the DEA method offer two basic advantages in the efficiency analysis [24]:

- First, it is a simple methodology that converts indicators into user-friendly efficiency parameters;
- Secondly, fewer variables are needed to calculate the indicators than to estimate the DEA Model. Therefore, even scarce data may prove advantageous in the estimation of the efficiency of the indicators.

This study includes 4 inputs and 3 outputs, with 31 DMUs to meet the required degrees of freedom. Based on the above principle and the results of principal component analysis, we selected the Health Institution, Health Professionals, Beds in Health Institution, Total Health Expenditure as input indicators; and Hospital Business Income, Outpatient Visits, and Bed Utilization Rate as output indicators.

### Methods to determine input/output indicators

#### Delphi method

For the Delphi method, we selected experts from public health, health management, hospital management and health department with a 100% response rate. The majority of the participants (20) were doctors (55%), including 25% women. The average age was 50 years. All the experts were briefed on the aim of the study and the Delphi procedure. The results of the standardized questionnaire were analyzed using descriptive statistics, such as, consensus, mean values, standard deviation and coefficient of variation, and returned to the experts. The descriptive data analysis for the second and third round was performed using SPSS 13.0 for Windows software (IBM). The study was conducted from May to July 2013.

A comprehensive literature review was carried out to ensure that all potential health resource indicators were considered. A draft list of 224 indicators was prepared. The edited list of 224 indicators was sent to each of the 20 experts in the Delphi panel for additional suggestions and comments. The revised draft list of 220 indicators (third-stage) included 4 first-stage indices and 10 second-stage indices. A final list of 170 indicators (third-stage) of health resources was established.

Due to the complexity of data analysis involving inequity in health resource allocation, it was difficult to include all the indicators from the factor analysis (FA) in the assessment. A new round of Delphi procedure was used to select candidate variables for FA. Based on the calculation of a comprehensive score of indicators and its representativeness, the Delphi panel recommended division of 18 indicators into input and output for FA from 170 indicators. The 9 input candidate variables (Health Institution, Beds in Health Institution, Health Professionals, Total Health Expenditure, Number of Health Professionals per 1000 Population, Number of Beds in Health Institution per 1000 Population, Average Medical Expense per Outpatient, and Average Medical Expense per Inpatient) were derived from 4 second-stage indices including “health institution resources”, “health beds”, “health professionals”, and “health funds”. The 9 output candidate variables (Outpatient Visits, Bed Utilization Rate, Hospital Business Income, Inpatients (Leave the hospital), Inpatients (Admitted to hospital), Stay Days, Average Stay Days in Hospitals, Daily Visits Per Doctor, Daily Inpatients Per Doctor) were derived from 2 second-stage indices including “population health efficiency”, “health service supply efficiency”.

#### Factor analysis (FA)

Factor Analysis (FA) and Principal Component Analysis (PCA) are two broad classes of procedures that are commonly used to reduce a set of observed variables to a set of new variables. Factor analysis is a statistical tool used to account for the observed correlations among several variables, particularly when “causation is complex and multivariate, and the basic concepts have been elusive” [25]. Principal Component Analysis is computed without regard to any underlying structure caused by latent variables. Components are calculated using all of the variance of the manifest variables, and all of that variance appears in the solutions [26].

In this paper, FA was mainly conducted to determine the key indicators for input/output from 18 candidate variables (9 input indicators and 9 output indicators). Health resource and service indictor data were prepared for FA by including variables that were believed to be related to each other, and that allowed sufficient observation to provide reliable estimation of correlations between the variables. PCA for factor extraction was chosen. The factors with the highest eigenvalues were considered as the most significant. Eigenvalues of ≥ 1.0 were considered significant [27].

Tables 1 and 2 show the corresponding component score and variance. With respect to the data on health resources and health services in 31 provinces in China, 4 input factors (Health Institution, Health Professionals, Beds in Health Institution, and Total Health Expenditure), and 3 output factors (Hospital Business Income, Outpatient Visits, and Bed Utilization Rate) suggest more than 96.5% of the total variance, respectively. Hence, 4 factors and 3 factors were considerably representative of health resource allocation and service utilization, and were used to evaluate the equity and efficiency of the process. Each indicator with a strong correlation coefficient value (> 80%) was considered as a significant parameter contributing to the indicators of health resource allocation and service utilization across the 31 provinces (Table 3).

**Table 1:** Total Variance Explaned.

Health resources (Input)				Health services (Output)			
Component	Eigenvalue	% of Variance	Cumulative	Component	Eigenvalue	% of Variance	Cumulative
**1**	8.112	90.135	90.135	**1**	8.086	89.841	89.841
**2**	0.428	4.761	94.895	**2**	0.402	3.426	93.267
**3**	0.253	2.813	97.708	**3**	0.391	3.138	96.405
**4**	0.117	0.669	98.523	**4**	0.136	1.69	98.095
**5**	0.105	0.633	99.01	**5**	0.102	1.002	99.097
**6**	0.046	0.508	99.518	**6**	0.059	0.658	99.755
**7**	0.034	0.375	99.893	**7**	0.022	0.245	100
**8**	0.006	0.07	99.963	**8**	0	0	100
**9**	0.003	0.037	100	**9**	0	0	100

**Table 2:** The factor loadings value of health resources and health services.

Health resources (Input)	Loadings value	Health services (Output)	Loadings value
**Health Institution**	**0.830**	**Outpatient Visits**	**0.888**
**Beds in Health Institution**	**0.886**	**Bed Utilization Rate**	**0.864**
**Health Professionals**	**0.898**	**Hospital Business Income**	**0.870**
**Total Health Expenditure**	**0.923**	Inpatients(Leave the hospital)	0.190
Number of Health Professionals per 1000 Population	0.097	Inpatients(Admitted to hospital)	0.190
Number of Beds in Health Institution per 1000 Population	0.084	Stay day	0.164
Average Medical Expense per Outpatient	0.125	Average Stay Days in Hospitals	-0.070
Average Medical Expense per Inpatient	0.114	Daily Visits Per Doctor	0.083
Total Assets	0.221	Daily Inpatients Per Doctor	0.120

**Table 3:** Health resources and health services of 31 provinces in China^a^.

	Health resources (Input)				Health services (Output)		
Provinces	Health Institution^b^	Beds in Health Institution	Health Professionals^c^	Total Health Expenditure (USD*10^4^)^d^	Outpatient visits (Times)	Bed Utilization Rate (%)	Hospital Business Income (USD*10^4^)
Beijing	9,495	94,735	181,936	1,522,225.52	161,728,305	84.4	1,280,145.51
Tianjin	4,428	49,423	73,318	530,117.04	86,906,788	90	473,560.59
Hebei	80,185	266,479	301,672	959,959.60	335,411,546	85.6	894,108.07
Shanxi	40,339	157,132	191,416	509,470.54	109,464,383	75.4	397,134.61
Inner Mongolia	22,908	100,633	131,603	416,397.69	87,800,819	81.1	327,529.70
Liaoning	35,229	215,815	235,623	878,117.60	154,400,472	85.2	780,036.18
Jilin	19,785	121,240	139,010	481,285.26	89,831,148	75	394,005.78
Helongjiang	21,749	165,255	195,029	637,137.96	105,729,867	79.6	532,924.39
Shanghai	4,740	107,130	140,740	1,349,487.22	210,629,305	96.6	1,186,770.39
Jiangsu	31,680	296,390	350,544	1,941,334.82	406,837,180	92.9	1,763,596.28
Zhejiang	30,515	194,759	306,922	1,794,354.69	408,862,250	94.6	1,591,535.85
Anhui	22,884	204,210	217,591	759,047.07	204,303,899	87.2	679,640.03
Fujian	27,147	124,232	158,791	637,599.53	175,181,563	90.6	584,375.25
Jiangxi	39,154	135,570	165,938	542,618.44	178,937,053	94.1	465,975.21
Shandong	68,275	416,148	481,738	1,697,765.72	517,942,116	85	1,541,900.53
Henan	76,128	349,612	396,300	1,127,098.88	464,965,269	88.1	1,043,063.03
Hubei	35,625	223,980	268,122	1,005,356.44	267,984,254	98.7	899,466.62
Hunan	59,634	257,687	282,511	960,951.74	216,852,772	94.8	883,543.07
Guangdong	45,930	325,038	485,585	2,473,097.52	652,532,340	86.6	2,252,891.98
Guangxi	34,026	152,039	204,011	640,694.42	205,285,704	93.3	569,657.57
Hainan	4,816	28,465	43,295	155,209.43	37,025,321	86.5	125,818.43
Chongqing	17,650	115,627	120,151	502,228.42	125,215,307	91.2	432,058.03
Sichuan	75,815	334,663	352,259	1,220,469.36	389,125,909	96.7	1,096,293.24
Guizhou	25,943	117,534	113,801	351,987.69	108,106,958	87.1	311,908.58
Yunnan	23,248	173,434	150,982	605,802.39	180,248,724	88.7	520,501.75
Tibet	6,602	9,592	10,782	38,075.46	10,502,419	69.6	16,768.55
Shaanxi	36,396	153,847	197,173	566,177.80	146,659,544	84	473,571.00
Gansu	26,632	94,907	105,908	303,626.45	107,000,739	82.1	221,937.98
Qinghai	5,887	23,117	27,520	91,429.59	20,826,836	77.3	67,787.62
Ningxia	4,132	25,805	31,983	121,056.09	28,123,628	91.1	92,217.28
Xinjiang	17,412	125,391	130,604	503,128.02	76,803,860	92.5	396,144.72

^a^ Data from China health statistics annual 2012.

^b^ Health Institution includes hospital, grassroots health care institution, specialized public health institution, other institution. Grassroots health care institution includes community health center & station, sub-district health center, village clinic, outpatient department, clinic. Specialized public health institution include CDC, specialized disease prevention & treatment Institution, health education center, MCH center, emergency center, center for blood collection & supply, center for health supervision, center for family planning service.

^c^ Health Professionals include practicing physicians, practicing physician assistant, registered nurses, pharmacists, inspection technician, image technician, health supervisors and trainee doctors, etc.

^d^ 1 USD = 6.2452CNY (13/5/2014).

## Results

### Equity evaluation based on concentration index and Gini coefficient

#### Concentration Index

The CI results were based on every province as an analytical unit, accounting for the 31 units (provinces) in the population. A concentration curve was drawn with the cumulative proportion of health inputs on the y-axis, and cumulative proportion of the population (31 units) ranked by economic status (beginning with the poorest and ending with the richest) on the x-axis. A general CI of the country reflected equity distribution of health inputs in the country.

Table 4 shows the results of our inequity analysis. The CIs reflecting inequity, were -0.116 (Health Institution), -0.012 (Beds in Health Institution), 0.038 (Health Professionals), and 0.111 (Outpatient Visits), which were statistically significant at 5%. These results indicate that the input “Health Professionals” was allocated higher income levels. Compared with the lower income levels, the higher income levels were more likely to utilize outpatient services in China. The negative value of the CI shows that Health Institution, Beds in Health Institution, and inpatient services were utilized by the lower-income population. These results indicate that the socioeconomic inequities observed in health resources allocation and health services utilization may lead to significant public health issues [28].

**Table 4:** Concentration index for health resources allocation and health services utilization.

	Health resources allocation			Health services utilization
	Health Institution	Beds in Health Institution	Health Professionals	Outpatient Visits
CI	-0.116	-0.012	0.038	0.111

#### Gini coefficient

The Gini coefficient is frequently used as an index of unequal health care resource distribution in the population. For accuracy, we collected data from subordinate units (district and counties) of each province to determine the population size and resource density (S2 Table). For each province, three Lorenz curves were drawn (Health Institution, Beds in Health Institution, Health Professionals) with the cumulative proportion of a health input on the y-axis, and cumulative proportion of the population (districts and counties in the province), ranked by economic status (beginning with the poorest and ending with the richest) on the x-axis. Three Gini coefficients were obtained for each province, analyzing equitable distribution of health inputs in the province. Finally, the 31 provinces were compared based on the results of the three health inputs.

**Fig 2:**
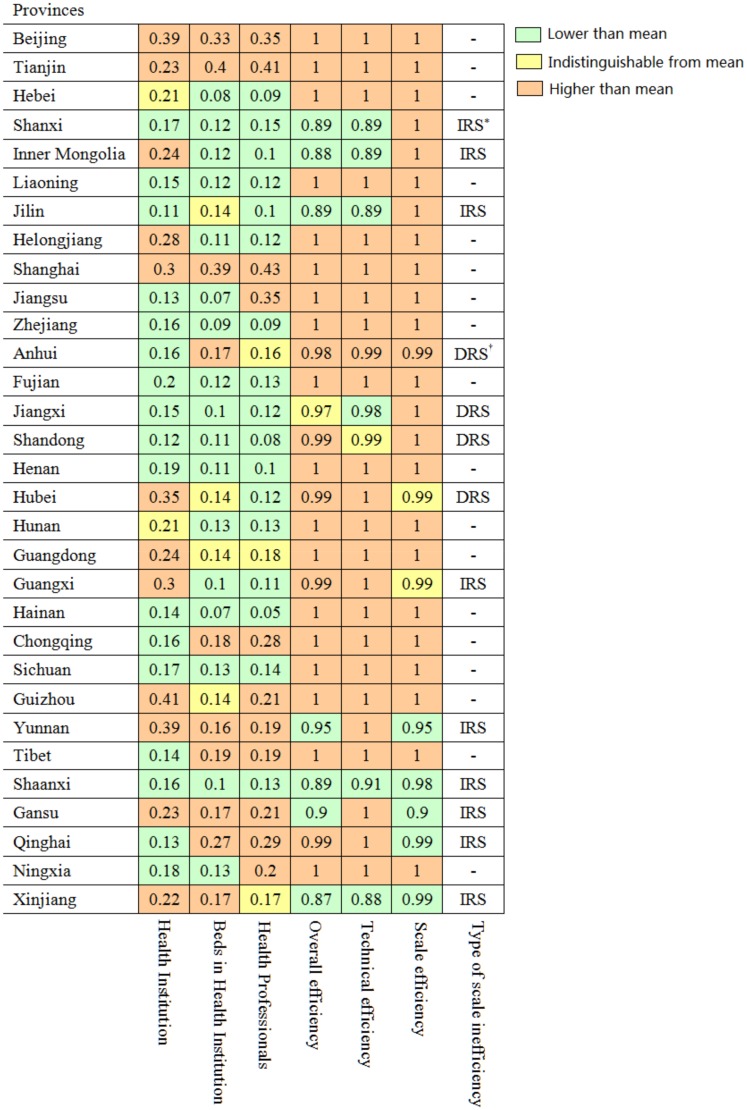
Gini coefficient, overall, technical and scale efficiency scores and returns to scale characteristics for health resources allocation and health services utilization of each province. Numbers in cells show the coefficient of each province for each index. * IRS: increasing return to scale. † DRS: decreasing return to scale.

Fig 2 illustrates the health resource allocation in the population of 31 provinces in China. The Gini coefficient for Health Institution in Guizhou (0.41) was more than four times higher than that of Jilin (0.11), indicating a substantial inequity in Health Institution allocation. For Beds in Health Institution, inequity of health allocation was found not only in developing areas but also in the largest cities. Provinces in the western region, including Qinghai, Tibet, and Xinjiang, showed high Gini coefficients ranging between 0.17 and 0.27. However, three of the four municipalities directly under the central government of China, namely, Tianjin, Shanghai, and Beijing, had the highest Gini coefficients (0.40, 0.39, and 0.33, respectively). The Gini coefficient for Health Professionals indicates inequity of allocation between provinces, with the Gini coefficient for Shanghai (0.43) more than eight times higher than that of Hainan (0.05). The detailed health resources in each province representing Gini coefficient are listed in S2 Table.

### Efficiency evaluation by data envelopment analysis (DEA)

#### Overall efficiency

Overall Efficiency was measured using the CRS model, which indicates that under similar circumstances, the actual output was similar to the ratio of maximum output. According to the VRS model, the OE is a product of TE and SE.

As shown in Fig 2, the average overall efficiency score of the 31 provinces was 0.973 in 2011. Based on the scores, 18 provinces (58.1%) were efficient in terms of health resource allocation, whereas the remaining 13 provinces (41.9%) were inefficient. Among the inefficient provinces, Xinjiang had the lowest score of 0.868, which indicates that its efficiency was 86.8% of that of the efficient provinces.

#### Pure technical efficiency and scale efficiency

Fig 2 shows the TE and SE scores and the returns to scale characteristics of the individual provinces. The mean scores of pure TE and SE in the 31 provinces were 0.981 and 0.992, respectively. Of the 31 provinces included in the study, 23 (74.2%) were technically efficient. The other 8 provinces were technically inefficient, with an average TE score of 0.927. This finding suggests that the 8 inefficient provinces potentially reduced their current input while maintaining their output levels unchanged. In other words, the 8 technically inefficient provinces produced more output at their current levels of input. Nineteen (61.3%) provinces had an SE score of 100% (Shandong province scored 99.9%, or approximately 100%), which implies that they had the most productive scale size for that particular input-output mix. The remaining 12 provinces were found to be scale-inefficient, with a mean SE score of 0.982. Anhui, Jiangxi and Hubei provinces had a decreasing scale ratio, suggesting that input size can be reduced without affecting their current output levels. On the other hand, Shanxi, Inn Mongolia, Jilin, Guangxi, Yunnan, Shaanxi, Gansu, Qinghai and Xinjiang provinces showed increasing scale benefit, suggesting that their scales of health resources allocation were inefficient, with a scope for strengthening their inputs.

#### Input-output analysis

An input-output analysis of 31 provinces with overall inefficiency was also performed to examine the redundant inputs and the expected outputs by measuring the distance between these provinces and their efficient peers [29]. Of the 12 scale-inefficient provinces, 9 (75.0%) had IRS and the remaining 3 (25.0%) had DRS. These findings indicate that 75.0% of the scale-inefficient provinces had operations that were too small and needed an expansion of their scale of operation. However, 25.0% of the inefficient provinces in China needed to scale down their operations to achieve CRS.

Compared with the efficient provinces (Fig 2), the inefficient provinces should either reduce or increase their inputs to enhance their efficiency (Table 5). The inefficient provinces need to reduce the average number of health staff by 38.4%, and the number of beds by 17.9%, to maintain constant levels of the current output. Alternatively, the inefficient provinces could increase the average number of outpatient visits by 9.9% and the bed utilization rate by 2.1%, at the current input levels.

**Table 5:** The excess of input and inadequate of output of inefficient provinces.

	Input				Output		
DMU	Health Institution	Beds in Hospital	Health Professionals	Total Health Expenditure	Outpatient Visits (Times)	Bed Utilization Rate (%)	Hospital Business Income
Shanxi	6908	17414	367774	0	29401040	3.059	0
Inn Mongolia	0	9425	11856	0	8785033	2.878	0
Jilin	0	22823	28671	0	13452901	1.867	0
Anhui	0	29922	58728	0	9349013	0.948	0
Jiangxi	0	11747	30128	0	11199022	1.586	0
Shandong	0	49158	100980	0	62345239	5.929	0
Hubei	0	19898	401981	0	11783229	0	0
Guangxi	0	15787	12675	0	8829042	2.334	0
Yunnan	0	17929	10779	0	9786390	0.894	0
Shaanxi	0	25806	44207	0	11134902	0.981	0
Gansu	4479	6223	2355	0	3922012	0	0
Qinghai	0	4473	3154	0	4528900	1.482	0

TE and SE are relative efficiency indices, which express the efficiency of health resources utilization in a province compared to that of the 30 other provinces. A 1% difference in health resource utilization efficiency suggests a huge resource surplus or shortage.

The results for Inner Mongolia (OE = 0.88, TE = 0.89, SE = 1), for example, indicate that the province should reduce its health staff to 11,856, and its beds to 9,425, to attain an OE at the present input level. In other words, to maintain the efficiency of excessive resource allocation, the number of outpatient visits needs to be increased to 8,785,033, and the bed utilization rate increased by 2.9%. The result of Inner Mongolia may be influenced by the scale of demographic and administrative area, but this estimation still provides a guideline for health resource allocation and service utilization.

## Discussion

The CI method enabled analysis of income-related inequity of health resource allocation and health service utilization in China [30, 31]. Our findings suggest inequitable resource allocation and service utilization among different population groups. Further, Health Professionals and Outpatient Visits are concentrated at higher income levels, while the Health Institutions and Beds in Health Institution are concentrated at the lower income levels. The findings suggested that the Outpatient Visits and Bed Utilization Rate were affected by inefficiency. For the inefficient provinces, there is a need to transfer their excessive input resources to grassroots health care institutions to meet the requirement for primary health services.

A few provinces showed good performance in terms of equity and efficiency. For example, Hainan, Shandong, and Zhejiang provinces showed efficient output of health services based on the input resources, thus reflecting an equitable allocation. The biggest eastern cities including Shanghai, Beijing, and Tianjin, had generally efficient but inequitable health resource allocation. In the central region, Jilin, Jiangxi, and Shannxi appeared to have had equitable resource allocation, but the efficiency of service outputs was inadequate. In the western region, including Yunnan, Qinghai, and Gansu, both resource allocation and service utilization were inequitable and inefficient. The overall factors such as the quality of resources, geographic location, and efficiency of primary health care services should be considered in any decision-making.

Health professionals are often attracted by monetary incentives and opportunities for career progression. Well-developed areas provide higher salaries and greater research opportunities. The CI also showed inequities in outpatient service utilization, indicating a disproportionate share of resources utilized by higher-income individuals. Compared with the affluent groups lower-income individuals are more likely to suffer from diseases that require more inpatient visits.

Affluent individuals tend to be afflicted with chronic diseases that require frequent outpatient visits on a long-term basis. In well-developed regions, such as eastern China, people have general health insurance coverage, which normally results in a high proportion of outpatient reimbursement. In economically underdeveloped areas, such as western China, accessibility to health resources is low. Residents suffering from common or chronic diseases fail to utilize outpatient services, resulting in inequity of outpatient service utilization.

In the present study, four main types of health institutions were included, i.e., hospitals, grassroots health care institutions, specialized public health institutions, and other institutions. Grassroots health care institutions account for 96.2% of the health institutions in China, whereas hospitals account for only 2.3%. Our findings suggest that even people with lower income are able to access health institutions. A potential explanation for this finding is that health institutions need government support for resource allocation. Therefore, resource allocation is not only affected by income level but also by government funding that enables access to primary health care equally by people with low incomes. Similarly, when planning the number of beds in a health institution, policy makers need to consider the population size.

Due to geographical conditions, people in developing regions often need to travel long distances in order to access hospital services. Low income is usually a barrier that prevents frequent hospital visits until hospitalization is inevitable. Therefore, the utilization of beds and inpatient services in health institutions is predominantly seen in the lower-income groups.

The average overall efficiency score of 0.8 shows that the 31 provinces included in the study produced similar output, while saving 20% on their input. Optimizing the input/output and controlling the scale of operations could improve the efficiency of resource allocation and service utilization in all the provinces. At their current scale, inefficient provinces are primarily tasked with developing human resources and improving medical skills and staff performance. Training for public health, rural health, and urban health professionals and technical personnel to optimize their management structures in order to effectively utilize inputs and improve the quality of health care services is needed.

Class III hospitals are representative of the high-quality health resources in China. Of the total number of hospitals in China, only 6.37% are graded as Class III, 46.68% of which are in the eastern region indicating that 30% of the provinces have 50% of the high-quality health resources. About 20% high-quality health resources cater to nearly 40% high-quality health services which is suggestive of quantitative and qualitative differences in resources and services across the provinces and hospitals.

Health services provided by primary health institutions are inefficient, and unable to meet the primary health care needs. No significant differences were observed between the primary, secondary and tertiary health care services in terms of health insurance currently. Patients with common health conditions visit specialty hospitals for better treatment, resulting in inequitable resource allocation and service utilization.

Several countries face similar basic challenges, which include the positioning of value orientation of health resource allocation; the governmental functional orientation to resource allocation, and the role of market forces. The USA and Sweden vary in their value orientation of resource allocation. The United States has the biggest proportion of medical investment in the world. It also has the most advanced medical technology and the highest total health expenditure and per capita health care cost as a percentage of total GDP. However, the population health is unsatisfactory. Life expectancy at birth in the USA was 71 years in 2012 [32].

The Swedish government allocates health resources as welfare services, with policies in place to reduce inequity in resource allocation. Sweden has a high quality of medical care, and the health status and average life expectancy of its population are among the best in the world. At the same time, the Swedish state monopoly has caused inefficiency in health resource allocation, and the "generous" health care expenditure has been exerting increasingly heavy pressure on the government finances. This approach has not only exceeded the capacity of the economy but has also increased the taxpayer’s burden. The Swedish government has thus adjusted the budget in accordance to the quality and effectiveness of health services, promoting efficient use of health services by encouraging competition among health institutions. These policies provide consumers with an increased freedom of choice in selecting the services. Based on an analysis of the inputs and outputs in the United States and Sweden, we suggest that governments should work according to the local conditions to enhance equity and efficiency.

From the discussion above, we have some suggestions to ameliorate the inequity in distribution of health resources in China. According to the regional economic development level, China should adopt a different configuration mode for health resource allocation and services utilization. For the relatively backward areas, focus should be on improving the equity of allocation of health resources. In addition commitment to meet the basic medical demand from the low-income groups is required. The eastern developed areas should aim to improve the efficiency of health resources allocation and service utilization while taking cognizance of the state of the economy to ensure provision of better quality health services.

Educating residents to utilize community health service centers for primary health care would boost the net utilization of services. The compensation ratio of medical insurance should lean towards the community health service centers and institutes. Policy should encourage ailing residents to utilize community health service centers, and seriously ill residents to attend hospitals. In addition, the government also needs to strengthen the establishment of grassroots medical institutions which will help reduce the observed redundancy in input levels and overall service utilization. Developing grassroots health facilities will help expand the availability and access to health services with relatively small investments. It is particularly important for the underdeveloped areas to bring about health equity.

Institutional measures are required to shape the structure of health resources: First, integrate the existing infrastructure for battling infectious diseases and develop a preventive focus. Second, strengthen the grassroots health infrastructure and its capacity for improved service delivery. Third, strengthen the support in terms of skill development and strengthening of key disciplines.

Cultivating talent and flow service mode: At present, China's high-income area accrues a large volume of health human resources, and at the same time its low-income area residents have poor access to quality medical services and inequitable distribution of diagnosis and treatment. The flow of health human resources can be resolved in two broad ways: by shifting of human resources from areas with a disproportionate excess of health human resources to the underserved areas. Another approach would be to build capacity of rural health human resources by institutionalizing training activities in developed regions. Return of these personnel to rural areas after a period of training will help improve the quality of medical care in the economic backward areas in a sustainable manner.

Major limitations of this study include inadequate representativeness of the indicators for evaluating equity and efficiency of health recourse allocation and health service utilization, which may have affected the comparison among provinces. However, these indicators have commonly been used in similar studies. Secondly, the data used may not represent the most current statistics, which reduces the timeliness of the decision-making. Furthermore, the methods of equity and efficiency evaluation were conducted independently in two phases, which needs improvement in the general analysis.

Further studies are needed with additional indicators to achieve a comprehensive evaluation of health resource allocation and service utilization based on improved accuracy and reliability of the data. A balanced model is needed to represent both the equity and efficiency of resource allocation and utilization on a global scale.

## Supporting Information

S1 Table Social and Economic Conditions of China. (DOCX)

S2 Table Health resources in each province for Gini coefficient. (DOC)
