# Supplementary material for: Study on Equity and Efficiency of Health Resources and Services Based on Key Indicators in China
Source: PLoS One. 2015 Dec 17;10(12):e0144809. doi: 10.1371/journal.pone.0144809 (PMC4683010; doi:10.1371/journal.pone.0144809)
Supplement: S1 Table — (DOCX) [file pone.0144809.s001.docx]

| **S1 Social and Economic Conditions of China** | | | | | | | |
| --- | --- | --- | --- | --- | --- | --- | --- |
| Region | Provinces | Land area | Population | GDP | Per capita GDP | Environment | Agriculture |
|  |  | (KM^2^×10^4^） | (1×10^4^) | (one hundred million USD) | (USD) |  |  |
| Eastern Region | Beijing | 1.68 | 2019 | 2619.47 | 13152.46 | Facing the ocean, flat terrain | Grain, aquatic product, oil, iron ore, salt and other resources |
|  | Tianjin | 1.18 | 1355 | 1830.85 | 13797.53 |  |  |
|  | Hebei | 18.77 | 7241 | 3963.73 | 5492.22 |  |  |
|  | Liaoning | 14.8 | 4383 | 3603.44 | 8228.92 |  |  |
|  | Shanghai | 0.63 | 2347 | 3140.42 | 13506.82 |  |  |
|  | Jiangsu | 10.26 | 7899 | 7951.66 | 10085.78 |  |  |
|  | Zhejiang | 10.18 | 5463 | 5235.22 | 9597.59 |  |  |
|  | Fujian | 12.14 | 3720 | 2848.31 | 7684.62 |  |  |
|  | Shandong | 15.7 | 9637 | 7432.22 | 7731.74 |  |  |
|  | Guangdong | 17.98 | 10505 | 8617.40 | 8228.26 |  |  |
|  | Hainan | 3.54 | 877 | 411.50 | 95.10 |  |  |
| Brief summary |  | 106.86 | 55446 | 47654.22 | 8872.82 |  |  |
| Central Region | Shanxi | 15.64 | 3593 | 1815.99 | 5067.35 | Located in the inland, plateau, hills and many plains | Grain, energy, various metals resources |
|  | Jilin | 18.74 | 2749 | 1722.82 | 6269.32 |  |  |
|  | Helongjiang | 46.00 | 3834 | 2045.62 | 5335.81 |  |  |
|  | Anhui | 13.96 | 5968 | 2472.05 | 4145.62 |  |  |
|  | Jiangxi | 16.69 | 4488 | 1895.11 | 4234.62 |  |  |
|  | Henan | 16.70 | 9388 | 4455.16 | 4741.29 |  |  |
|  | Hubei | 18.59 | 5758 | 3205.61 | 5583.83 |  |  |
|  | Hunan | 21.18 | 6596 | 3212.32 | 4879.86 |  |  |
| Brief summary |  | 167.50 | 42374 | 20824.68 | 5032.21 |  |  |
| Western Region | Inner Mongolia | 118.30 | 2482 | 2330.66 | 9409.45 | Vast, higher ground, complex terrain, plateau, desert, grassland and basin | Asperity and dry, not suitable for crop growth |
|  | Guangxi | 23.67 | 4645 | 1916.48 | 4141.53 |  |  |
|  | Chongqing | 8.24 | 2919 | 1637.82 | 5644.20 |  |  |
|  | Sichuan | 48.50 | 8050 | 3439.97 | 4275.36 |  |  |
|  | Guizhou | 17.62 | 3469 | 932.81 | 2685.17 |  |  |
|  | Yunnan | 39.40 | 4631 | 1431.66 | 3101.37 |  |  |
|  | Tibet | 120.22 | 303 | 99.11 | 3284.60 |  |  |
|  | Shaanxi | 20.58 | 3743 | 2027.22 | 5422.03 |  |  |
|  | Gansu | 45.40 | 2564 | 818.08 | 3192.98 |  |  |
|  | Qinghai | 72.23 | 568 | 267.44 | 4726.57 |  |  |
|  | Ningxia | 5.18 | 639 | 337.15 | 5299.33 |  |  |
|  | Xinjiang | 166.49 | 2209 | 1059.23 | 4825.55 |  |  |
| Brief summary |  | 685.83 | 36222 | 16297.63 | 4667.35 |  |  |
