# Supplementary material for: Study on Equity and Efficiency of Health Resources and Services Based on Key Indicators in China
Source: PLoS One. 2015 Dec 17;10(12):e0144809. doi: 10.1371/journal.pone.0144809 (PMC4683010; doi:10.1371/journal.pone.0144809)
Supplement: S2 Table — (DOC) [file pone.0144809.s002.doc]

**S2 Health resources in each province for Gini coefficient**

| Province |  | Population (10^4^) | Health Institution | Beds in Health Institution | Health Professionals |
| --- | --- | --- | --- | --- | --- |
| **Beijing** |  |  |  |  |  |
|  | Dongcheng District | 91.0 | 547 | 10022 | 28970 |
|  | Chaoyang District | 365.8 | 1201 | 16107 | 45653 |
|  | Fengtai District | 217.0 | 502 | 7999 | 17735 |
|  | Shijingshan District | 63.4 | 196 | 3515 | 7217 |
|  | Haidian District | 340.2 | 547 | 10022 | 32532 |
|  | Mentougou District | 29.4 | 711 | 4693 | 10042 |
|  | Fangshan District | 96.7 | 255 | 2653 | 4332 |
|  | Tongzhou District | 125.0 | 1006 | 6152 | 9881 |
|  | Shunyi District | 91.5 | 593 | 2593 | 8224 |
|  | Changping District | 173.8 | 502 | 3252 | 7005 |
|  | Daxing District | 142.9 | 849 | 7982 | 11325 |
|  | Huairou District | 37.1 | 453 | 1534 | 3774 |
|  | Pinggu District | 41.8 | 309 | 1972 | 4455 |
|  | Miyun District | 47.1 | 672 | 1440 | 4389 |
|  | Yanqing District | 31.9 | 323 | 1048 | 2517 |
|  | Xicheng District | 124.0 | 616 | 13831 | 37657 |
| **Tianjin** |  |  |  |  |  |
|  | Baodi District | 79.92 | 335 | 1772 | 2817 |
|  | Beichen District | 66.91 | 173 | 1318 | 3129 |
|  | Binhai New Area | 248.21 | 568 | 6199 | 13755 |
|  | Dongli District | 57.01 | 133 | 1200 | 2247 |
|  | Heping District | 27.35 | 123 | 5790 | 13204 |
|  | Hebei District | 78.85 | 198 | 3708 | 6961 |
|  | Hedong District | 86.09 | 207 | 3232 | 6408 |
|  | Hexi District | 87.06 | 236 | 7652 | 12636 |
|  | Hongqiao District | 53.15 | 115 | 2828 | 5513 |
|  | Ji County | 78.48 | 580 | 1646 | 4423 |
|  | Jinnan District | 59.31 | 251 | 1594 | 4226 |
|  | Jinghai County | 64.70 | 405 | 1390 | 3427 |
|  | Nankai District | 101.82 | 198 | 5637 | 11592 |
|  | Ninghe County | 43.10 | 177 | 1091 | 2301 |
|  | Wuqing District | 94.95 | 535 | 2747 | 5571 |
|  | Xiqing District | 68.47 | 197 | 1619 | 1962 |
| **Hebei** |  |  |  |  |  |
|  | Baoding | 1127.2 | 2284 | 35113 | 48312 |
|  | Cangzhou | 719.8 | 1144 | 25332 | 33372 |
|  | Chengde | 348.9 | 1174 | 14308 | 19007 |
|  | Handan | 923.9 | 1075 | 33282 | 38737 |
|  | Hengshui | 436.4 | 571 | 13279 | 18033 |
|  | Langfang | 440.0 | 934 | 15879 | 22808 |
|  | Qinhuangdao | 300.6 | 670 | 13896 | 17532 |
|  | Shijiazhuang | 1028.0 | 2427 | 40811 | 58254 |
|  | Tangshan | 762.7 | 1767 | 35001 | 47030 |
|  | Xingtai | 715.6 | 1023 | 23472 | 27851 |
|  | Zhangjiakou | 437.4 | 1786 | 16531 | 20386 |
| **Shanxi** |  |  |  |  |  |
|  | Datong | 3339733 | 1215 | 15639 | 25271 |
|  | Jincheng | 2285526 | 858 | 8574 | 17603 |
|  | Jinzhong | 3270025 | 1102 | 12631 | 22550 |
|  | Linfen | 4344986 | 1167 | 16546 | 28846 |
|  | Lvliang | 3751846 | 709 | 10948 | 20818 |
|  | Shuoyang | 1725617 | 375 | 6447 | 9689 |
|  | Taiyuan | 4235352 | 2553 | 29876 | 51065 |
|  | Xinzhou | 3085039 | 780 | 11448 | 22180 |
|  | Yangquan | 1374242 | 460 | 7270 | 12613 |
|  | Yuncheng | 5166767 | 1837 | 24921 | 37236 |
|  | Changzhi | 3353632 | 948 | 14159 | 25428 |
| **Inner Mongolia** |  |  |  |  |  |
|  | Alxa League | 23.53 | 153 | 958 | 1854 |
|  | Bayannur | 166.33 | 1541 | 7923 | 12011 |
|  | Baotou | 269.29 | 2030 | 13590 | 21498 |
|  | Chifeng | 431.93 | 998 | 17527 | 24701 |
|  | Ordos | 199.93 | 1746 | 7191 | 13108 |
|  | Hohhot | 291.19 | 1815 | 13037 | 22993 |
|  | Hulun Buir | 253.97 | 1961 | 12411 | 21960 |
|  | Tongliao | 313.64 | 4208 | 10098 | 19915 |
|  | Wuhai | 54.14 | 332 | 3093 | 4275 |
|  | Ulanqab | 213.46 | 682 | 5904 | 8924 |
|  | Xilin Gol League | 103.31 | 535 | 3219 | 6298 |
|  | Xing'an League | 160.99 | 510 | 5591 | 9209 |
| **Liaoning** |  |  |  |  |  |
|  | Anshan | 351.6 | 1740 | 20357 | 27069 |
|  | Benxi | 154.3 | 318 | 9556 | 13578 |
|  | Chaoyang | 341.0 | 1306 | 11609 | 20065 |
|  | Dalian | 588.5 | 2357 | 34395 | 52122 |
|  | Dandong | 241.1 | 501 | 13637 | 16568 |
|  | Fushuan | 220.1 | 693 | 11831 | 15650 |
|  | Fuxin | 192.1 | 528 | 7593 | 13877 |
|  | Huludao | 281.3 | 908 | 13699 | 17243 |
|  | Jinzhou | 308.3 | 721 | 11944 | 16760 |
|  | Liaoyang | 182.4 | 621 | 11335 | 12427 |
|  | Panjin | 131.2 | 649 | 7056 | 11136 |
|  | Shenyang | 722.7 | 1814 | 44634 | 69333 |
|  | Tieling | 304.9 | 1164 | 8603 | 19517 |
|  | Yingkou | 235.5 | 883 | 10722 | 15394 |
| **Jilin** |  |  |  |  |  |
|  | Baicheng | 230.0 | 1553 | 7045 | 12204 |
|  | Baishan | 128.1 | 1100 | 7831 | 10021 |
|  | Jinlin | 441.0 | 3544 | 21313 | 33163 |
|  | Liaoyuan | 117.7 | 697 | 5587 | 8879 |
|  | Siping | 340.0 | 2101 | 13169 | 23031 |
|  | Songyuan | 290.0 | 2701 | 6486 | 15577 |
|  | Tonghua | 227.2 | 1801 | 10347 | 14692 |
|  | Korean Autonomous Prefecture of Yanbian | 217.0 | 2140 | 9908 | 16891 |
|  | Changchun | 767.7 | 4153 | 39712 | 58594 |
| **Heilongjiang** |  |  |  |  |  |
|  | Daqing | 290.6 | 761 | 13855 | 23029 |
|  | Da Hinggan Mountains | 51.2 | 274 | 2633 | 4378 |
|  | Harbin | 1064.2 | 1654 | 52439 | 71640 |
|  | Hegang | 105.9 | 554 | 6682 | 9173 |
|  | Heihe | 167.5 | 420 | 7470 | 9821 |
|  | Jixi | 186.3 | 567 | 9362 | 12498 |
|  | Jiamusi | 255.3 | 672 | 11156 | 16941 |
|  | Mudanjiang | 280.0 | 749 | 12600 | 19652 |
|  | Qitaihe | 92.1 | 186 | 3697 | 4974 |
|  | Qiqihar | 537.0 | 772 | 19550 | 25864 |
|  | Shuangyashan | 146.3 | 724 | 7724 | 10249 |
|  | Suihua | 542.1 | 610 | 12290 | 19424 |
|  | Yichun | 114.9 | 713 | 5944 | 8458 |
| **Shanghai** |  |  |  |  |  |
|  | Baoshan District | 193.5 | 249 | 5221 | 8316 |
|  | Chongming County | 72.5 | 115 | 3433 | 4470 |
|  | Fengxian District | 110.3 | 72 | 4568 | 4910 |
|  | Hongkou District | 85.2 | 139 | 6356 | 10071 |
|  | Huangpu District | 68.0 | 234 | 10241 | 19219 |
|  | Jiading District | 150.6 | 217 | 3088 | 5784 |
|  | Jinshan District | 75.9 | 112 | 3873 | 5491 |
|  | Jing'an District | 24.4 | 123 | 5237 | 10528 |
|  | Minhang District | 248.4 | 279 | 6926 | 12017 |
|  | Pudong New Area | 517.5 | 594 | 15374 | 24439 |
|  | Putuo District | 129.7 | 148 | 5467 | 7829 |
|  | Qingpu District | 111.8 | 118 | 2376 | 4444 |
|  | Songjiang District | 165.0 | 158 | 4077 | 6587 |
|  | Xuhui District | 109.5 | 288 | 13480 | 22007 |
|  | Yangpu District | 132.4 | 164 | 7296 | 10192 |
|  | Zhabei District | 83.8 | 121 | 5202 | 7349 |
|  | Changning District | 69.0 | 227 | 4915 | 10327 |
| **Jiangsu** |  |  |  |  |  |
|  | Changzhou | 464.97 | 1100 | 16936 | 26808 |
|  | Huaian | 480.34 | 2116 | 16002 | 27073 |
|  | Lianyungang | 438.61 | 2666 | 12991 | 26442 |
|  | Nanjing | 810.91 | 2268 | 34503 | 62114 |
|  | Nantong | 728.91 | 3283 | 29058 | 43501 |
|  | Suzhou | 1051.87 | 2856 | 42972 | 63402 |
|  | Taizhou | 462.6 | 1998 | 16106 | 26969 |
|  | Wuxi | 643.22 | 1938 | 28780 | 39403 |
|  | Suqian | 476.64 | 3131 | 15306 | 27782 |
|  | Xuzhou | 857.26 | 4415 | 32960 | 54799 |
|  | Yancheng | 723.74 | 3059 | 22927 | 38157 |
|  | Yangzhou | 446.3 | 1982 | 16909 | 27120 |
|  | Zhenjiang | 313.43 | 868 | 10940 | 18248 |
| **Zhejiang** |  |  |  |  |  |
|  | Hangzhou | 873.8 | 4142 | 45291 | 79292 |
|  | Huzhou | 289.9 | 1359 | 10599 | 19474 |
|  | Jiaxing | 453.1 | 1364 | 16272 | 27423 |
|  | Jinhua | 538.6 | 4000 | 17558 | 29305 |
|  | Lishui | 211.6 | 1685 | 8566 | 13596 |
|  | Ningbo | 762.8 | 4221 | 27127 | 55424 |
|  | Quzhou | 211.9 | 2157 | 8134 | 12501 |
|  | Shaoxing | 493.4 | 2887 | 16849 | 27739 |
|  | Taizhou | 599.9 | 3061 | 17536 | 36660 |
|  | Wenzhou | 914.3 | 4973 | 22783 | 52810 |
|  | Zhoushan | 113.7 | 666 | 4044 | 8584 |
| **Anhui** |  |  |  |  |  |
|  | Anqing | 530.6 | 2361 | 16067 | 27170 |
|  | Pengpu | 317.6 | 1355 | 13853 | 18994 |
|  | Chizhou | 141.4 | 878 | 4536 | 8211 |
|  | Chuzhou | 393.1 | 1453 | 12009 | 18988 |
|  | Fuyang | 761.9 | 2331 | 22075 | 35683 |
|  | Haozhou | 610.0 | 1534 | 10900 | 19547 |
|  | Hefei | 752.1 | 2107 | 34251 | 47885 |
|  | Huaibei | 211.5 | 678 | 11166 | 15104 |
|  | Huainan | 233.1 | 1175 | 11736 | 15663 |
|  | Huangshan | 134.9 | 1077 | 5614 | 8398 |
|  | Lu'an | 563.6 | 2510 | 14842 | 25095 |
|  | Ma'anshan | 218.8 | 814 | 6486 | 12370 |
|  | Tongling | 73.1 | 296 | 4186 | 5783 |
|  | Wuhu | 356.6 | 1339 | 14004 | 20805 |
|  | Suzhou | 536.6 | 1759 | 12761 | 23025 |
|  | Xuancheng | 254.7 | 1217 | 8556 | 12793 |
| **Fujian** |  |  |  |  |  |
|  | Fuzhou | 720 | 2040 | 26707 | 48283 |
|  | Longyan | 256 | 591 | 12261 | 15412 |
|  | Nanping | 265 | 458 | 10569 | 13850 |
|  | Ningde | 283 | 582 | 9780 | 13663 |
|  | Putian | 279 | 308 | 8656 | 12389 |
|  | Quanzhou | 821 | 950 | 21606 | 27034 |
|  | Sanming | 251 | 800 | 10748 | 13905 |
|  | Xiamen | 361 | 965 | 11533 | 25044 |
|  | Zhangzhou | 484 | 591 | 11924 | 16193 |
| **Jiangxi** |  |  |  |  |  |
|  | Fuzhou | 393.8 | 441 | 7829 | 13217 |
|  | Ganzhou | 842.8 | 1393 | 22874 | 29250 |
|  | Jian | 484.3 | 642 | 13179 | 18130 |
|  | Jingdezhen | 159.9 | 348 | 5592 | 7945 |
|  | Jiujiang | 476.3 | 787 | 16526 | 22411 |
|  | Nanchang | 508.9 | 717 | 20876 | 34580 |
|  | Pingxiang | 186.8 | 334 | 6896 | 11688 |
|  | Shangrao | 662.4 | 1051 | 18454 | 25447 |
|  | Xinyu | 114.7 | 226 | 4393 | 6300 |
|  | Yichun | 545.3 | 811 | 16629 | 22141 |
|  | Yingtan | 113.4 | 371 | 3264 | 5208 |
| **Shandong** |  |  |  |  |  |
|  | Binzhou | 377.1 | 1949 | 18852 | 29083 |
|  | Dezhou | 560.3 | 3448 | 17524 | 32872 |
|  | Dongying | 205.5 | 1644 | 10761 | 17241 |
|  | Heze | 828.8 | 2871 | 27142 | 53781 |
|  | Jinan | 688.5 | 4460 | 34920 | 58590 |
|  | Jining | 812.9 | 6602 | 32743 | 55464 |
|  | Laiwu | 130.6 | 1212 | 5588 | 10124 |
|  | Liaocheng | 584.6 | 4965 | 19667 | 32995 |
|  | Linyi | 1009.1 | 6586 | 39904 | 62652 |
|  | Qingdao | 879.5 | 7436 | 39980 | 64232 |
|  | Rizhao | 281.9 | 2017 | 10606 | 17502 |
|  | Taian | 551.4 | 3821 | 23863 | 41528 |
|  | Weihai | 280.1 | 2239 | 17308 | 22454 |
|  | Weifang | 915.5 | 6582 | 41024 | 78678 |
|  | Yantai | 697.6 | 5309 | 38481 | 52384 |
|  | Zaozhuang | 375.3 | 2193 | 13778 | 22652 |
|  | Zibo | 455.6 | 4941 | 24007 | 37396 |
| **Henan** |  |  |  |  |  |
|  | Anyang | 515 | 6736 | 19227 | 34346 |
|  | Hebi | 158 | 2150 | 6024 | 11562 |
|  | Jiaozuo | 353 | 3082 | 15403 | 25373 |
|  | Kaifeng | 466 | 2830 | 16827 | 31713 |
|  | Luoyang | 657 | 3849 | 28873 | 46849 |
|  | Luohe | 255 | 1749 | 9531 | 17571 |
|  | Nanyang | 1013 | 6511 | 30314 | 56300 |
|  | Pingdingshan | 492 | 4110 | 21033 | 35196 |
|  | Puyang | 356 | 5327 | 13210 | 25172 |
|  | Sanmenxia | 224 | 1961 | 9459 | 15621 |
|  | Shangqiu | 736 | 7490 | 22474 | 46799 |
|  | Xinxiang | 566 | 6169 | 24609 | 43051 |
|  | Xinyang | 611 | 4051 | 15881 | 32774 |
|  | Xuchang | 430 | 4480 | 13691 | 30370 |
|  | Zhengzhou | 886 | 4044 | 52750 | 74813 |
|  | Zhoukoudian | 895 | 7681 | 24659 | 50691 |
|  | Zhumadian | 709 | 3396 | 22527 | 39596 |
| **Hubei** |  |  |  |  |  |
|  | Ezhou | 105.10 | 458 | 3800 | 5847 |
|  | Tujia and Miao Autonomous Prefecture of Enshi | 329.74 | 976 | 13869 | 14762 |
|  | Huanggang | 621.04 | 612 | 16371 | 29230 |
|  | Huangshi | 243.46 | 337 | 10303 | 16515 |
|  | Jingmen | 287.99 | 617 | 10889 | 14038 |
|  | Jingzhou | 570.40 | 242 | 16625 | 28452 |
|  | Shiyan | 334.81 | 816 | 17840 | 24572 |
|  | Suizhou | 216.99 | 1090 | 6000 | 10977 |
|  | Wuhan | 1002.00 | 2848 | 59540 | 61564 |
|  | Xianning | 246.79 | 266 | 7772 | 14339 |
|  | Xiangfan | 552.72 | 494 | 19791 | 30162 |
|  | Xiaogan | 482.49 | 424 | 13281 | 22193 |
|  | Yichang | 406.85 | 1164 | 17338 | 27713 |
| **Hunan** |  |  |  |  |  |
|  | Changde | 573.3 | 1407 | 20667 | 25880 |
|  | Chenzhou | 460.5 | 972 | 19104 | 23400 |
|  | Hengyang | 716.6 | 632 | 23921 | 32465 |
|  | Huaihua | 475.1 | 1123 | 23117 | 22943 |
|  | Loudi | 379.3 | 509 | 11596 | 15577 |
|  | Shaoyang | 710.7 | 726 | 20699 | 25392 |
|  | Xiangtan | 276.5 | 921 | 11711 | 16187 |
|  | Yiyang | 431.4 | 804 | 13737 | 18088 |
|  | Yongzhou | 521.3 | 923 | 17525 | 21169 |
|  | Yueyang | 548.5 | 1304 | 17294 | 23166 |
|  | Zhangjiajie | 149.0 | 336 | 5932 | 7103 |
|  | Changsha | 709.1 | 2680 | 47036 | 64297 |
|  | Zhuzhou | 388.1 | 1247 | 17914 | 23148 |
| **Guangdong** |  |  |  |  |  |
|  | Chaozhou | 268.37 | 809 | 5358 | 8737 |
|  | Dongguan | 825.48 | 1006 | 22814 | 44574 |
|  | Foshan | 723.10 | 1086 | 24526 | 42989 |
|  | Guangzhou | 1275.14 | 2363 | 65940 | 122411 |
|  | Heyuan | 298.18 | 303 | 8457 | 12577 |
|  | Huizhou | 463.36 | 809 | 13092 | 23802 |
|  | Jiangmen | 446.55 | 740 | 14247 | 22665 |
|  | Jieyang | 591.54 | 260 | 9844 | 15890 |
|  | Maoming | 588.26 | 419 | 17046 | 25003 |
|  | Meizhou | 426.81 | 1202 | 11633 | 20978 |
|  | Qingyuan | 373.80 | 658 | 11097 | 15240 |
|  | Shantou | 541.71 | 394 | 13057 | 20772 |
|  | Shanwei | 295.50 | 310 | 6561 | 10248 |
|  | Shaoguan | 285.00 | 718 | 13007 | 18793 |
|  | Shenzhen | 1046.74 | 2481 | 24079 | 71976 |
|  | Yangjiang | 244.49 | 384 | 7437 | 11289 |
|  | Yunfu | 237.92 | 306 | 6160 | 9816 |
|  | Zhanjiang | 706.92 | 1028 | 22360 | 31034 |
|  | Zhaoqing | 395.14 | 735 | 10889 | 20012 |
|  | Zhongshan | 314.23 | 473 | 10698 | 19211 |
|  | Zhuhai | 156.76 | 478 | 6736 | 14227 |
| **Guangxi** |  |  |  |  |  |
|  | Baise | 349.46 | 2452 | 11786 | 19272 |
|  | Beihai | 155.44 | 440 | 5304 | 7554 |
|  | Chongzuo | 201.14 | 1286 | 5447 | 7980 |
|  | Fangchenggang | 87.84 | 539 | 2573 | 5444 |
|  | Guigang | 415.67 | 534 | 9373 | 20711 |
|  | Guilin | 478.82 | 1661 | 15691 | 33023 |
|  | Hechi | 339.34 | 564 | 10823 | 17378 |
|  | Hezhou | 197.03 | 1433 | 4883 | 10709 |
|  | Laibin | 211.82 | 124 | 6385 | 10975 |
|  | Liuzhou | 379.39 | 2151 | 16454 | 28560 |
|  | Nanning | 673.40 | 2324 | 28821 | 48680 |
|  | Qinzhou | 310.96 | 442 | 9374 | 15476 |
|  | Wuzhou | 290.85 | 1677 | 9441 | 17641 |
|  | Yulin | 553.84 | 3122 | 15684 | 27668 |
| **Hainan** |  |  |  |  |  |
|  | Haikou | 209.73 | 502 | 10155 | 20538 |
|  | Sanya | 70.55 | 297 | 2615 | 5457 |
| **Chongqing** |  |  |  |  |  |
|  | Banan District | 93.47 | 624 | 3961 | 空缺 |
|  | Beibei District | 72.1 | 438 | 3480 | 空缺 |
|  | Bishan County | 59.57 | 390 | 1814 | 空缺 |
|  | Chengkou County | 19.03 | 161 | 815 | 空缺 |
|  | Dadukou District | 31.58 | 181 | 1148 | 空缺 |
|  | Dazu District | 72.97 | 409 | 3065 | 空缺 |
|  | Dianjiang County | 70.1 | 370 | 2546 | 空缺 |
|  | Fengdu County | 63.95 | 434 | 2235 | 空缺 |
|  | Fengjie County | 81.93 | 479 | 2642 | 空缺 |
|  | Fuling County | 108.36 | 629 | 4342 | 空缺 |
|  | Hechuan District | 131.25 | 546 | 3480 | 空缺 |
|  | Jiangbei District | 77.66 | 342 | 4436 | 空缺 |
|  | Jiangjin District | 124.93 | 624 | 4781 | 空缺 |
|  | Jiulongpo | 111.63 | 557 | 5374 | 空缺 |
|  | Kai County | 116.08 | 612 | 3952 | 空缺 |
|  | Liangping County | 68.39 | 573 | 2045 | 空缺 |
|  | Nan'an District | 78.98 | 319 | 2413 | 空缺 |
|  | Nanchuan District | 53.79 | 297 | 2470 | 空缺 |
|  | Pengshui Tujia and Miao Autonomous County | 54.06 | 436 | 1366 | 空缺 |
|  | Qijiang District | 107.59 | 602 | 4447 | 空缺 |
|  | Qianjiang District | 44.63 | 235 | 2600 | 空缺 |
|  | Rongchang County | 66.69 | 437 | 2440 | 空缺 |
|  | Shapingba District | 104.35 | 344 | 4626 | 空缺 |
|  | Shizhu Tujia Autonomous County | 41.14 | 271 | 1690 | 空缺 |
|  | Tongliang County | 60.1 | 835 | 2414 | 空缺 |
|  | Tongnan County | 64.25 | 415 | 1670 | 空缺 |
|  | Wanzhou District | 157.22 | 1217 | 7252 | 空缺 |
|  | Wushan County | 48.99 | 376 | 1154 | 空缺 |
|  | Wuxi County | 40.95 | 306 | 905 | 空缺 |
|  | Wulong County | 34.85 | 278 | 1292 | 空缺 |
|  | Xiushan Tujia and Miao Autonomous County | 49.75 | 287 | 1239 | 空缺 |
|  | Yongchuan District | 104.44 | 510 | 4559 | 空缺 |
|  | Youyang Tujia and Miao Autonomous County | 57.46 | 341 | 1823 | 空缺 |
|  | Yubei District | 138.64 | 433 | 3492 | 空缺 |
|  | Yuzhong District | 63.9 | 352 | 8835 | 空缺 |
|  | Yunyang County | 91.11 | 546 | 3176 | 空缺 |
|  | Changshou District | 78.29 | 504 | 3123 | 空缺 |
|  | Zhong County | 74.82 | 950 | 2525 | 空缺 |
| **Sichuan** |  |  |  |  |  |
|  | Ngawa Tibetan and Qiang Autonomous Prefecture | 90.22 | 1562 | 3292 | 5670 |
|  | Bazhong | 329.63 | 3062 | 9868 | 16297 |
|  | Chengdu | 1407.08 | 7401 | 79780 | 130490 |
|  | Dazhou | 548.56 | 4063 | 16913 | 27917 |
|  | Deyang | 359.19 | 2816 | 15725 | 22030 |
|  | Tibetan Autonomous Prefecture of Garzê | 110.00 | 2359 | 3280 | 7617 |
|  | Guang'an | 321.00 | 3347 | 9828 | 15575 |
|  | Guangyuan | 249.00 | 3285 | 12392 | 17356 |
|  | Leshan | 324.33 | 3026 | 14764 | 20093 |
|  | Yi Autonomous Prefecture of Liangshan | 454.10 | 5047 | 13410 | 20196 |
|  | Luzhou | 422.50 | 4778 | 15277 | 22940 |
|  | Meishan | 295.83 | 1989 | 10462 | 15805 |
|  | Mianyang | 462.00 | 4173 | 22236 | 29964 |
|  | Nanchong | 628.53 | 8269 | 21883 | 33008 |
|  | Neijiang | 370.91 | 3126 | 13744 | 19350 |
|  | Panzhihua | 121.99 | 1025 | 7972 | 11272 |
|  | Suining | 326.01 | 3724 | 11468 | 17245 |
|  | Ya'an | 151.71 | 1367 | 8027 | 9092 |
|  | Yinbin | 446.00 | 4137 | 18006 | 24507 |
|  | Ziyang | 363.01 | 4912 | 15067 | 21204 |
|  | Zigong | 268.40 | 2346 | 11757 | 17485 |
| **Guizhou** |  |  |  |  |  |
|  | Anshun | 228 | 1761 | 6491 | 9282 |
|  | Bijie | 654 | 634 | 17175 | 22527 |
|  | Guiyang | 439 | 2919 | 24347 | 36551 |
|  | Liupanshui | 285 | 1458 | 10412 | 14207 |
|  | Tongren | 309 | 244 | 8536 | 13857 |
|  | Zunyi | 610 | 901 | 20710 | 30353 |
| **Yunnan** |  |  |  |  |  |
|  | Baoshan | 252.5 | 376 | 7812 | 7224 |
|  | Yi Autonomous Prefecture of Chuxiong | 270.4 | 609 | 11437 | 13791 |
|  | Bai Autonomous Prefecture of Dali | 347.8 | 690 | 12400 | 14671 |
|  | Dai-Jingpo Autonomous Prefecture of Dehong | 122.1 | 341 | 4543 | 6256 |
|  | Deqen Zang Autonomous Prefecture | 40.3 | 260 | 935 | 2067 |
|  | Hani-Yi Autonomous Prefecture of Honghe | 453.5 | 1009 | 19295 | 20150 |
|  | Kunming | 648.6 | 3103 | 41386 | 54470 |
|  | Lijiang | 125.4 | 108 | 3646 | 3046 |
|  | Lincang | 244.8 | 341 | 5862 | 5765 |
|  | Lisu Autonomous Prefecture of Nujiang | 53.6 | 84 | 1559 | 1654 |
|  | Puer | 254.3 | 421 | 6878 | 10745 |
|  | Qujing | 589.9 | 605 | 19472 | 15117 |
|  | Zhuang-Miao Autonomous Prefecture of Wenshan | 354.2 | 372 | 9519 | 12416 |
|  | Dai Autonomous Prefecture of Xishuangbanna | 114.2 | 760 | 5340 | 6436 |
|  | Yuxi | 231.8 | 186 | 10494 | 9537 |
|  | Zhaotong | 525.9 | 462 | 12772 | 10053 |
| **Tibet** |  |  |  |  |  |
|  | Ngari Prefecture | 9.7 | 74 | 631 | 642 |
|  | Qamdo Prefecture | 66.6 | 243 | 1447 | 1799 |
|  | Lhasa | 56.3 | 239 | 2195 | 4195 |
|  | Nyingchi Prefecture | 19.8 | 131 | 895 | 1032 |
|  | Nagqu Prefecture | 46.8 | 203 | 1303 | 1521 |
|  | Xigaze Prefecture | 71.0 | 323 | 2220 | 2378 |
|  | Shannan Prefecture | 33.2 | 167 | 951 | 1428 |
| **Shaanxi** |  |  |  |  |  |
|  | Xi'an | 851.34 | 5554 | 41010 | 79999 |
|  | Tongchuan | 83.82 | 972 | 4629 | 7691 |
|  | Baoji | 372.72 | 2788 | 17211 | 25199 |
|  | Xianyang | 491.23 | 4649 | 19764 | 37157 |
|  | Weinan | 530.49 | 3893 | 15913 | 30422 |
|  | Yan'an | 219.40 | 3419 | 8962 | 17119 |
|  | Hanzhong | 341.51 | 3951 | 14580 | 23816 |
|  | Yulin | 335.24 | 5198 | 14390 | 24139 |
|  | Ankang | 263.07 | 3189 | 8598 | 14298 |
|  | Shangluo | 233.62 | 2616 | 7998 | 14519 |
| **Gansu** |  |  |  |  |  |
|  | Baiyin | 171.33 | 379 | 6171 | 8712 |
|  | Dingxi | 270.51 | 542 | 9052 | 10744 |
|  | Tibetan Autonomous Prefecture of Ganan | 68.85 | 252 | 2044 | 4505 |
|  | Jiayuguan | 23.32 | 107 | 1464 | 2616 |
|  | Jinchang | 46.59 | 396 | 1910 | 3578 |
|  | Jiuquan | 110.07 | 433 | 5059 | 7198 |
|  | Lanzhou | 362.09 | 1606 | 25411 | 33448 |
|  | Hui Autonomous Prefecture of Linxia | 196.29 | 666 | 6396 | 7503 |
|  | Longnan | 256.77 | 1948 | 6086 | 13282 |
|  | Pingliang | 207.67 | 1164 | 7853 | 11519 |
|  | Qingyang | 221.48 | 777 | 6355 | 10549 |
|  | Tianshui | 327.47 | 726 | 10526 | 15334 |
|  | Wuwei | 181.97 | 620 | 6946 | 9446 |
|  | Zhangye | 120.46 | 453 | 5835 | 7856 |
| **Qinghai** |  |  |  |  |  |
|  | Tibetan Autonomous Prefecture of Golog | 18.56 | 89 | 521 | 826 |
|  | Tibetan Autonomous Prefecture of Haibei | 27.30 | 94 | 1109 | 1313 |
|  | Haidong Prefecture | 139.70 | 360 | 3319 | 3939 |
|  | Tibetan Autonomous Prefecture of Hainan | 44.17 | 138 | 1876 | 1659 |
|  | Mongolia Tibetan Autonomous Prefecture of Haixi | 38.00 | 153 | 1702 | 2609 |
|  | Tibetan Autonomous Prefecture of Huangnan | 25.67 | 65 | 862 | 1011 |
|  | Xining | 121.18 | 592 | 13078 | 18600 |
|  | Tibetan Autonomous Prefecture of Yushu | 28.31 | 110 | 704 | 1079 |
| **Ningxia** |  |  |  |  |  |
|  | Guyuan | 115.32 | 1233 | 4362 | 5921 |
|  | Shizuishan | 74.00 | 566 | 3791 | 6199 |
|  | Wuzhong | 127.00 | 883 | 4221 | 6771 |
|  | Yinchuan | 203.00 | 862 | 10329 | 18366 |
|  | Zhongwei | 118.12 | 599 | 3179 | 4581 |
| **Xinjiang** |  |  |  |  |  |
|  | Aksu Prefecture | 265.4 | 1308 | 11771 | 11852 |
|  | Altay Prefecture | 61.3 | 770 | 3112 | 5164 |
|  | Monggolian Autonomous Prefecture of Bayingolin | 149.7 | 1212 | 8136 | 10779 |
|  | Mongolian Autonomous Prefecture of Bortala | 52.6 | 639 | 2643 | 4233 |
|  | Hui Autonomous Prefecture of Changji | 173.4 | 1392 | 9224 | 12730 |
|  | Hami Prefecture | 59.2 | 467 | 3471 | 5449 |
|  | Hotan Prefecture | 210.1 | 1503 | 10875 | 10473 |
|  | Kashi Prefecture | 398.0 | 3463 | 18282 | 21448 |
|  | Karamay | 65.6 | 6 | 93 | 1838 |
|  | Kirgiz Autonomous Prefecture of Kizilsu | 58.3 | 300 | 3140 | 3848 |
|  | Tacheng Prefecture | 145.6 | 1210 | 4493 | 7416 |
|  | Turpan Prefecture | 61.2 | 365 | 2262 | 3922 |
|  | Urumqi | 330.7 | 1772 | 26530 | 39986 |
|  | Kazak Autonomous Prefecture of Ili | 286.2 | 1976 | 13886 | 19428 |
